# The Impact of Screen Exposure on Screen Addiction and Sensory Processing in Typically Developing Children Aged 6–10 Years

**DOI:** 10.3390/children11040464

**Published:** 2024-04-13

**Authors:** Yasin Tekeci, Berkan Torpil, Onur Altuntaş

**Affiliations:** 1 Occupational Therapy Department, Faculty of Gülhane Health Sciences, University of Health Sciences Turkey, Ankara 06018, Turkey; berkan.torpil@sbu.edu.tr; 2 Occupational Therapy Department, Faculty of Health Sciences, Hacettepe University, Ankara 06018, Turkey; onur.altuntas@hacettepe.edu.tr

**Keywords:** children, typical development, screen addiction, screen exposure, sensory processing

## Abstract

As technology continues to develop, children are spending more time in front of screens, which can lead to significant problems. For children aged 5 years and above, screen time of 2 or more hours per day on average is considered problematic. This study aimed to investigate the impact of screen exposure on screen addiction and sensory processing in typically developing children aged 6–10 years. The study analyzed 74 children who had a screen exposure time of 2 h or more and 71 children who had a screen exposure time of less than 2 h. The Dunn Sensory Profile was used to evaluate sensory processing skills, and the Problematic Media Use Scale was used to measure screen addiction. The group with high screen exposure showed statistically significant differences in screen addiction, distraction, and sedentary factors (*p* < 0.05). No significant differences were found in other parameters. Based on these findings, it has been determined that excessive screen exposure leads to a more sedentary lifestyle, increased screen addiction, and distraction in typically developing children aged 6–10 years. It is important to consider the duration of screen exposure in typically developing children aged 6–10 years and to conduct further studies on this topic.

## 1. Introduction

The introduction of television technology into our lives and the emergence of computer and internet technology in the following years, followed by the increase in portable screen-based devices such as smartphones and tablets, have caused significant changes in the lives of almost all humanity worldwide [1,2]. In addition, the increase in the use of smartphones and tablets and the increase in social media environments have also caused many changes in contemporary life [1,2,3]. With the more intensive use of technology such as smartphones, televisions, and tablets, the time spent in front of the screen has increased, especially in children and adolescents, and the effects of this situation on health need to be considered more (3). The time spent watching television, DVDs, videos, or playing computer or video games, using smartphones and tablets, and using social media (commonly known as screen time) has been associated with various serious health consequences, such as impaired language acquisition, violent behaviors, tobacco use, and obesity in children. It is important to determine the effects of screen use on human health and to limit screen time and establish safe screen time to prevent the negative effects of screen exposure [2].

Various studies have been conducted on the appropriate screen usage time, and in 2001, the American Academy of Pediatrics provided important information on limiting children’s screen time [3,4]. The Academy recommends that children under 2 years of age should not spend any time in front of screens, meaning that they should not be exposed to screens. For children aged 2–5, screen time should be limited to one hour per day. It has been stated that for children aged 5 and above, healthy screen time should be less than 2 h. It is important to follow these guidelines to promote healthy screen habits [4]. Despite the recommendations of the American Academy of Pediatrics, it has been noted that the time spent in front of screens is increasing every day [5]. Additionally, research on screen usage age in children has found that it has decreased from four years to four months since the 1970s [6].

With the advancement of technology, production technology has advanced and made access to electronic devices easier. The increased availability of electronic devices has increased the use of smartphones, tablets, computers, and televisions, and concerns have arisen regarding the increase in screen exposure time, especially in children and adolescents, and the increase in this duration in children [7,8,9,10,11,12]. The fact that electronic devices such as smartphones, tablets, televisions, and computers that cause screen exposure have become an integral part of daily activities can lead to increased screen time and even uncontrolled screen exposure, especially in children. Unfortunately, this shows that exposure to screens often begins in early infancy [13,14,15]. Research shows that children spend a significant portion of their waking hours, approximately 8 h per day, engaged in screen-based activities. On the other hand, research shows that currently more than a third of children in developed countries have their own smartphone or tablet device [16,17,18]. Additionally, research has shown that young children are among the most frequent users of digital media applications [19].

Researchers observed that children who spend more than one hour a day in front of screens in early childhood and more than two hours a day after the age of five experience different health problems and various daily life activity problems. It is important to limit the time spent in front of the screen to protect and improve children’s physical, cognitive, sensory, and psychosocial health. Studies have found that increased screen time is linked to a variety of negative health outcomes and behavioral problems. These problems include unhealthy eating habits, obesity, poor sleep quality, and sleep problems, orthopedic problems, cardiovascular diseases, increased emotional dysregulation, decreased positive social behaviors, increased inattention and hyperactivity, low language skills, executive dysfunctions, cognitive problems, low physical activity and sensory processes. To reduce these risks, it is important to limit screen time, control screen use, and understand the negative effects of screen exposure time on children [5,6].

Different studies have been conducted on the effects of screen exposure time on children. In 2020, Dadson et al. conducted a study to investigate the relationship between screen time and sensory processing skills in children. The results showed a significant negative correlation between screen time and visual, tactile, body awareness, balance, planning, and general sensory processing skills [20]. The study found that increased screen time was associated with poorer sensory processing skills in these areas. Concerning the increasing screen exposure time of children at an early age, it has been suggested that it may lead to a range of issues, including decreased cognitive abilities, poor school performance, slowed growth, the development of addictive behaviors, poor sleep patterns, and increased obesity. These problems have been linked to sensory processing issues. It is important to note that these claims are not universally accepted, and further research is needed to fully understand the impact of screen time on children’s development [21]. We think it is important to investigate the effects of screen time on children in order to better determine the rapidly developing technology and its effects on our lives. We think that it is important to investigate both screen addiction and sensory processing skills, especially to see the effects of both behavioral and developmental aspects. According to our research, we did not find a study comparing screen addiction and sensory profile in 6–10-year-old typically developing children with and without normal screen usage time.

This study aims to compare the screen addiction and sensory processing skills of typically developing children aged 6–10 years with and without problematic screen exposure.

Our working hypothesis is as follows:

H0: There is no difference in screen addiction and sensory processing skills between children aged 6–10 years who show typical development and are exposed to screen time of 2 h or more compared to those exposed to less than 2 h of screen time. H0: There is no difference in screen addiction and sensory processing skills between children aged 6–10 years who show typical development and are exposed to screen time of 2 h or more compared to those exposed to less than 2 h of screen time.

## 2. Materials and Methods

This study was created in a case–control design, and typically developing children between the ages of 6 and 10 years were included in the current study. The study was conducted in accordance with the Declaration of Helsinki and with the permission of the scientific research ethics committee of a public university. Ethical approval of the study was obtained from the non-interventional clinical research ethics committee of a public university. All participants provided signed consent forms before the study.

### 2.1. Participants

The sample calculation was made with the G*Power (Version 3.1.9.7, University of Düsseldorf, Düsseldorf, Germany) program. The study’s sample size required a minimum of 65 participants in each group and a total of at least 130 participants. A total of 165 typically developing children aged 6–10 years were invited to this study. The study’s inclusion and exclusion criteria are presented below.

The inclusion criteria were (1) being between the ages of 6–10 years, (2) parental age between 18–65 years, (3) volunteering to participate in the study, (4) active participation of the child in the educational process, and (5) literacy of the parent.

Exclusion criteria were determined as (1) the child having a diagnosed disease or suspicion of diagnosis that may affect the study, and (2) the parent having any chronic disease that may affect the study.

The study data were collected from participants who applied to the pediatric unit of the occupational therapy department of a public university. The average daily total screen time of their children was obtained from activities such as smartphone and tablet use and television viewing during the day. The study was completed by 145 participants who met the inclusion and exclusion criteria. The American Academy of Pediatrics and most studies worldwide recommend limiting intensive screen use to 2 h [22,23,24,25,26]. Following the specified time limit for the screen, 145 participants were divided into two groups: participants who used screens for 2 or more hours (study group) and participants who used screens for less than 2 h (control group). Out of 145 participants, 74 were assigned to the study group and 71 to the control group. All participants will complete a sociodemographic data form, providing information such as age and gender, as well as the Dunn Sensory Profile to evaluate sensory processing [27] and the Problematic Media Use Scale to assess screen addiction [28].

### 2.2. Instruments

The Dunn Sensory Profile was developed by Dunn and colleagues to objectively assess children’s responses to common sensory experiences. Parents rate their child’s frequency of response to 125 experiences using a Likert scale. The experiences are divided into eight categories: The language is formal and free from grammatical errors, spelling mistakes, and punctuation errors. Auditory, Visual, Activity Level, Taste/Smell, Body Position, Movement, Touch, and Emotional/Social. The sensory profile was considered suitable for 3–10 children and standardized on 1200 children with and without disabilities. Content and construct validity were established. Responses were summarized in six sensory processing domains: Auditory Processing, Visual Processing, Vestibular Processing, Tactile Processing, Multisensory Processing, and Oral Sensory Processing. Additionally, five modulation processes and three domains of behavior and emotional responses were identified [29]. In 2015, a validity and reliability study was conducted on the Turkish version of this questionnaire. The study showed that Cronbach’s α values ranged from 0.63 to 0.97 for all subsections, and test–retest reliability was excellent (intraclass correlation coefficient > 0.90) [30]. The findings indicated that the Turkish version of the Sensory Profile can be used validly and reliably. However, normative data for Turkish children was not provided in this study [27].

The Problematic Media Use Scale was developed by Domoff et al. in 2017 to assess the problematic media use of children aged 4–11 years. It evaluates the potential addiction aspect of problematic media use through a parental report. The items of the scale were created based on the criteria for Online Gaming Addiction Disorder expressed in DSM V. The scale consists of a 27-item form that is scored using a Likert-type scale, with each item scored between 1 (never) and 5 (always). The total score for the Problematic Media Use Scale is calculated by averaging the scores obtained from all items. High scores on the scale indicate problematic use. The scale, completed by parents based on their observations of their child’s behavior, does not provide information about problematic use of a specific media tool. Rather, it aims to identify problematic use of visual media tools, such as phones, tablets, computers, and televisions, in general—in other words, screen addiction. The long form of the scale had Cronbach’s alpha values of 0.97 [31]. Furuncu and Öztürk conducted a validity study of the scale in 2020 [28].

### 2.3. Statistical Analyses

Data were analyzed with the IBM SPSS Statistics version 28.0 (IBM Corporation, Armonk, NY, USA) statistical software package program. Data are presented as mean ± standard deviation. The normality of data distributions was analyzed with the Kolmogorov–Smirnov test. Differences between groups were analyzed with a chi-square test for nominal data. Since the data did not show a normal distribution, non-parametric statistical methods were used in the study. Comparisons between the groups were analyzed by the Mann–Whitney U test. The power of the study was determined to be 80% with a statistical significance level of *p* < 0.05.

## 3. Results

The study found no significant difference in gender distribution between the groups. The study included a total of 145 participants, with 71 in the control group and 74 in the study group. The control group consisted of 30 females and 41 males, while the study group consisted of 32 females and 42 males. Both groups had a similar profile in terms of the number of siblings and school attendance. There was no difference in the school years of both groups. The sociodemographic data of the participants are presented in Table 1.

Upon examination of the sensory processing skills and screen addiction of both the study and control groups, it was found that the study group exhibited significantly higher levels of sedentary behavior (*p* < 0.05), distractibility (*p* < 0.01), and screen addiction (*p* < 0.001) compared to the control group. Table 2 presents the data on participants’ screen addiction and sensory processing skills for each group, as well as the differences in screen addiction and sensory processing between the groups.

## 4. Discussion

This study aims to compare the screen addiction and sensory processing skills of typically developing children aged 6–10 years with and without problematic screen exposure. Our findings support the existing literature, which suggests that the recommended maximum screen time for this age group is 2 h less per day. The study revealed that children who had a screen exposure time of two hours or more had a higher potential for screen addiction. Additionally, they exhibited more attention problems and sedentary behavior compared to those with normal screen exposure time. No significant differences were found between the groups when examining other sensory parameters. A detailed discussion of our findings from the current study is presented below.

The authors reported that the duration of screen use among typically developing children in the 6–10 age group has gradually increased in recent years [32]. The study found that children in this age group spend more time on screens than younger children but less time than older children [32]. It is important to consider the potential impact of excessive screen time on children’s development and wellbeing. The researchers also noted a gradual increase in screen time with age [32]. Other studies have shown that increased screen time is associated with negative social, psychological, physical, and neurological effects. Screen exposure has been identified as a significant public health concern [33,34]. The American Academy of Pediatrics conducted a study on safe screen time for different age groups. According to their findings, babies should not be exposed to screens during the first 2 years of life. Children between the ages of 2 and 6 should have a maximum of 1 h of screen time per day, while those between 6 and 10 should have a maximum of 2 h [4]. Exceeding these limits may result in significant developmental problems. In the current study, we compared screen addiction and sensory development in typically developing children aged 6–10 years who had less than 2 h of screen time with those who had 2 or more hours of screen time. The study findings indicate that children who spend 2 h or more on screens have a significantly higher risk of screen addiction compared to the control group. As screen time increases, children’s participation in activities of daily living may decrease, and screen addiction may arise, with activities such as using phones, computers, tablets, and watching television becoming more meaningful to them. It is important to limit screen time and encourage a variety of activities for children. The intense visual and auditory stimuli and continuously changing content of screen exposure may contribute to the development of addiction. Another possible reason for the increase in screen addiction could be the fact that children are spending more time at home due to the COVID-19 pandemic, leading to an increase in screen time. It is important to note that this is just one possible explanation, and further research is needed to fully understand the causes of screen addiction in children. This may be causing children to continue their excessive screen time habits even after the pandemic period has ended. In addition to the important benefits of technology, we think that this study is important in terms of understanding the negative effects of uncontrolled technology use, such as excessive screen exposure, on typically developing children aged 6–10 and provides important results to the literature. We think that it is important for researchers, clinicians, and parents working in this age group to take into consideration the duration of screen exposure in terms of the child’s general development and the healthy maintenance of activities of daily living.

Attention is a crucial aspect of cognitive function and can significantly impact other cognitive skills [24,35,36]. Studies have shown that increased screen time in children and young people can lead to obesity/adiposity, higher energy intake, behavioral problems, anxiety, hyperactivity, and attention issues [33,37,38]. Another study highlighted the limited information available on the precise impact of screen time and digital media on cognitive skills and attention [39]. Additionally, researchers found that attention is negatively affected by increased screen time, but controlled use can have a positive impact on attention [24]. This study found that children with more screen time experience higher levels of distraction. This supports the literature’s suggestion that controlling screen time and content can have a positive effect. It can be concluded that excessive screen time leads to increased distraction, possibly due to the child’s exposure to intense visual and auditory stimuli. This may result in reduced reactivity to other environmental stimuli. Intense content changes in the digital environment may cause children to pay less attention to other activities with fewer stimuli in their daily lives. Therefore, it is important to control the use of platforms that increase screen exposure, such as television, social media, the internet, tablets, and smartphones, in accordance with the values specified by the American Academy of Pediatrics. On the other hand, children with more screen exposure time have more distractions, which may lead to problems in the effective maintenance of activities of daily living and academic skills at school in children aged 6–10 years. Therefore, we think it is important to investigate activities of daily living and academic skills at school in these children.

The researchers found that increased screen time is associated with decreased physical activity levels in children [40,41,42]. This sedentary behavior can interfere with the movement patterns required for activities of daily living. The authors reported that a sedentary lifestyle is a significant public health issue and may lead to various metabolic disorders, such as obesity [42]. In another study, researchers found that children who spend more than two hours on screen time are more physically inactive than those who spend less time on screens [41]. Similarly, the current study found that children who spend two or more hours on screens have lower levels of physical activity than those who spend less than two hours on screens. Decreased participation in physical activities, that is, a more sedentary life, can be attributed to the increase in screen time in children. This may be because time spent in front of a screen leaves less time for other physical activities. In addition, screen addiction caused by increased time spent in front of the screen may also contribute to children’s disinterest in physical activities. Another reason is that intense stimuli created by technological devices that cause social screen exposure, such as a fast flow, an immersive effect, and the desire not to stay away from interaction on social media, may also limit participation in physical activities. We think that it is important to conduct studies on this situation, as decreased physical activity may negatively affect the general development of children. We think that it should not be forgotten that general negative health conditions such as diabetes and obesity, which can be caused by a sedentary life, may also pose a risk for these children in the future.

There are several limitations to this study. The first limitation is that the diversity of screen types such as television, tablet, and smartphone that constitute screen exposure is not known. Another limitation is that it is not known which content constitutes screen time. The reporting of screen time is a sensitive issue. Due to the variety and availability of electronic media devices, reports for parents or children may be inaccurate in both quantity and quality. One potential limitation is that screen time is based on parental observations, which can be influenced by factors such as family-child interaction or parental awareness of the situation, potentially affecting screen time or safe screen use. Another limitation was the inability to compare groups based on the age and gender of the participants.

## 5. Conclusions

Screen addiction and sensory processing skills were compared in typically developing children between the ages of 6 and 10 years who were exposed to intense screen time and those who were not, this gave us three important results. The first important result is screen addiction. It was determined that children with 2 h or more of screen time had more screen addiction than children with less screen time. Our second important result is that there are more attention deficits in typically developing children aged 6–10 years who have more screen time. The third important result is that typically developing children aged 6–10 years who have more screen exposure time lead a more sedentary life. We think that these results provide important results in showing families, researchers, and clinicians what effects excessive screen exposure will have on typically developing children aged 6–10 years. We think it is important for researchers and clinicians working in this age group to consider screen exposure time in their studies.

## Figures and Tables

**Table 1:** Demographic characteristics of the groups.

	**Control Group** **(*n* = 71)**		**Study Group** **(*n* = 74)**		***p***
	***n***	**%**	***n***	**%**	
**Sex**					>0.05
Female	30	42.3	32	43.2	
Male	41	57.7	42	56.8	
	**Mean ± SD** **(Minimum–Maximum)**		**Mean ± SD** **(Minimum–Maximum)**		
**Age**	7.94 ± 1.18 (6–10)		8.31 ± 1.21 (6–10)		>0.05
Number of Siblings	1.08 ± 0.78 (0–3)		1.20 ± 0.85 (0–3)		>0.05
School Time	3.77 ± 1.68 (1–7)		4.04 ± 1.63 (1–7)		>0.05

Note: Statistically significant values = *p* < 0.05.

**Table 2:** Comparison of screen time and sensory processing skills of the groups.

	Control Group (*n* = 71) Mean ± SD (Minimum–Maximum)	Study Group (*n* = 74) Mean ± SD (Minimum–Maximum)	*p*
**PMUS**	48.78 ± 18.80 (28–108)	70.08 ± 23.80 (28–126)	<0.001 *
**DUNN S.P.**			
Auditory processing	33.08 ± 7.49 (8–40)	31.45 ± 7.52 (8–40)	>0.05
Visual processing	39.53 ± 7.72 (9–45)	38.10 ± 9.05 (9–45)	>0.05
Vestibular processing	48.09 ± 9.41 (11–55)	46.95 ± 11.30 (11–55)	>0.05
Touch processing	88.14 ± 14.86 (23–100)	86.06 ± 19.09 (24–100)	>0.05
Multisensory processing	31.16 ± 5.53 (7–35)	29.12 ± 7.43 (7–35)	>0.05
Oral sensory processing	49.33 ± 10.83 (12–60)	48.08 ± 12.63 (12–60)	>0.05
Sensory processing related to endurance/tone	41.00 ± 6.85 (9–45)	38.31 ± 9.41 (9–45)	>0.05
Modulation related to body position and movement	38.92 ± 7.36 (9–45)	37.05 ± 9.43 (9–45)	>0.05
Modulation of movement affecting activity level	28.91 ± 5.74 (7–35)	26.81 ± 7.46 (7–35)	>0.05
Modulation of sensory input affecting emotional responses and activity level	16.35 ± 3.54 (4–20)	15.62 ± 4.11 (4–20)	>0.05
Modulation of visual input affecting emotional responses and activity level	17.84 ± 3.16 (4–20)	17.00 ± 4.21 (4–20)	>0.05
Emotional/social responses	70.11 ± 13.76 (17–85)	67.28 ± 17.65 (17–85)	>0.05
Behavioral outcomes of sensory processing	25.45 ± 5.09 (6–30)	23.77 ± 6.88 (6–30)	>0.05
Items indicating thresholds for response	12.80 ± 2.91(3–15)	12.87 ± 3.22 (3–15)	>0.05
**DUNN S.P. FACTORS**			
Registration	67.26 ± 10.98 (15–75)	62.52 ± 15.37 (15–75)	>0.05
Seeking	107.02 ± 22.59 (26–130)	104.37 ± 25.85 (26–130)	>0.05
Sensitivity	85.49 ± 15.14 (20–100)	82.37 ± 19.24 (20–100)	>0.05
Avoiding	127.46 ± 22.28 (30–150)	122.41 ± 28.13 (30–150)	>0.05
Sensation seeking	70.67 ± 14.46 (17–85)	68.39 ± 16.85 (17–85)	>0.05
Emotional reactive	65.28 ± 13.25 (16–80)	62.83 ± 16.98 (16–80)	>0.05
Low endurance/tone	41.00 ± 6.85 (9–45)	38.31 ± 9.41 (9–45)	>0.05
Oral sensory sensitivity	35.78 ± 8.81 (9–45)	35.17 ± 9.99 (9–45)	>0.05
Inattention/distractibility	29.00 ± 6.42 (7–35)	26.71 ± 7.01 (7–35)	<0.01 *
Poor registration	36.02 ± 6.05 (8–40)	34.58 ± 8.02 (8–40)	>0.05
Sensory sensitivity	17.29 ± 3.53 (4–20)	16.72 ± 4.45 (4–20)	>0.05
Sedentary	17.33 ± 3.65 (4–20)	15.68 ± 4.76 (4–20)	<0.05 *
Fine motor/perceptual	13.25 ± 2.56 (3–15)	12.09 ± 3.48 (3–15)	>0.05

Note: * Statistically significant values (*p* < 0.05) are shown in bold. **PMUS:** Problematic Media Use Scale. **DUNN S.P.:** Dunn Sensory Profile.

## Data Availability

The dataset analysed in this study are available on request from Yasin Tekeci (yasintekeci@gmail.com) due to (both ethical and privacy).
